# Regulating epithelial-mesenchymal plasticity from 3D genome organization

**DOI:** 10.1038/s42003-024-06441-w

**Published:** 2024-06-20

**Authors:** Qing You Pang, Yi-Chia Chiu, Ruby Yun-Ju Huang

**Affiliations:** 1 Neuro-Oncology Research Laboratory, National Neuroscience Institute, Singapore, 308433 Singapore; 2 School of Medicine, College of Medicine, National Taiwan University, Taipei, 10051 Taiwan; 3 Center for Advanced Computing and Imaging in Biomedicine, National Taiwan University, Taipei, 10051 Taiwan; 4 Department of Obstetrics & Gynaecology, Yong Loo Lin School of Medicine, National University of Singapore, Singapore, 119077 Singapore

**Keywords:** Cancer genomics, Epigenetics, Epithelial-mesenchymal transition

## Abstract

Epithelial-mesenchymal transition (EMT) is a dynamic process enabling polarized epithelial cells to acquire mesenchymal features implicated in development and carcinoma progression. As our understanding evolves, it is clear the reversible execution of EMT arises from complex epigenomic regulation involving histone modifications and 3-dimensional (3D) genome structural changes, leading to a cascade of transcriptional events. This review summarizes current knowledge on chromatin organization in EMT, with a focus on hierarchical structures of the 3D genome and chromatin accessibility changes.

## Abstract (web summary)

A Mini Review summarizing the current understanding of epithelial-mesenchymal transition (EMT) and 3D genome organization, focusing on the role of chromatin conformation and accessibility changes during the transitional phases of EMT.

## Introduction

### The paradigm shift of EMT from a binary to a spectral perspective

**Fig. 1:**
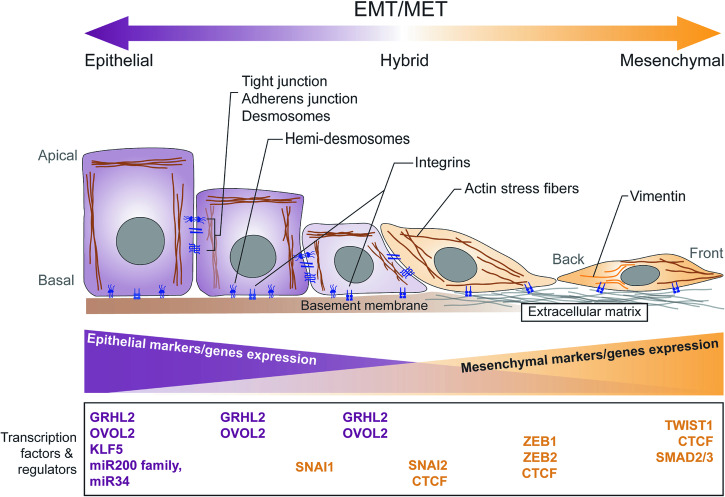
Cells at different phases of the EMT spectrum display varying combinations and degrees of cellular features associated with epithelial or mesenchymal states. Epithelial cells form connections with one another through different types of junctions, including adherens junctions, desmosomes, gap junctions, and tight junctions. The proper organization these cell-cell adhesion in epithelial cells is guided by apical-basal polarity. The epithelial cells are connected to the underlying basement membrane via hemidesmosomes, which contains integrin that facilitates binding to the basement membrane and is also linked to cytokeratins within the cell. Mesenchymal cells lack functional epithelial junctions and exhibit back-front polarity in their actin stress fibers. Vimentin-based intermediate filaments are present in mesenchymal cells, and they use integrin-containing focal adhesions to attach to the extracellular matrix. Some of the transcription factors and regulators known to regulate the different phases of the epithelial-to-mesenchymal transition are shown below the spectrum (epithelial regulators – purple, and mesenchymal regulators, orange).

Epithelial-mesenchymal transition (EMT) is a cellular process enabling epithelial cells to lose their epithelial features and adopt distinct mesenchymal phenotypes facilitating cell invasion and migration. EMT occurs in both physiological conditions such as gastrulation and wound healing and pathological conditions such as tissue fibrosis and cancer progression. The conventional view of EMT emphasizes on the transition in between two broadly defined states^1,2^. The “epithelial” and the “mesenchymal” states are viewed from their cellular features such as apical-basal polarity, cell-cell adhesion junctions, cell-matrix interactions, and their functional consequences in the migratory and invasive behaviour. These two broadly defined traits have established the binary concept of EMT and the reversibility during transition. Core regulatory networks that encompass a repertoire of EMT transcription factors and its downstream effector have been known to govern the binary EMT process^3^. Recently, the concept of EMT has undergone a major paradigm shift to the notion of multiple intermediate states of hybrid phenotypes as an EMT Spectrum^4–6^ with epithelial-mesenchymal plasticity (EMP) governing the convertibility among these states^5^ (Fig. 1). These in-between states have more dynamic and complex ‘mix-and-match’ of the above-mentioned cellular features. The understanding of how plasticity is regulated along the entire EMT spectrum thus becomes more challenging. For instance, the detailed transcriptional programme guiding the plasticity in between the various states within the EMT spectrum is less clear. Recent data has suggested that there might be transcriptional regulatory network switches to trigger the sequential transition during carcinoma progression^7^. A hierarchical regulatory landscape between 46 (co)transcription factors and 13 miRNAs was identified to be critically required for EMT in the non-tumorigenic mouse mammary epithelial cells NMuMG^8^. But what has been of contention is whether these transcriptional repertoires are context specific^9^ that there might not be universal master transcription regulators particularly in scenarios with known diverse heterogeneity such as tissue fibrosis and cancer metastasis.

### Multi-scale regulations of EMT

With the context-dependent nature of EMT regulation in mind, there is a need to go beyond transcriptional programs and examine the regulatory landscape of EMT at the genomic level. Additionally, integrating different perspectives is crucial to enable a broad and in-depth approach for a more comprehensive understanding. In a recent updated guideline for EMT research^5^, the official academic society for the EMT field, The EMT International Association (TEMTIA), recommended that EMT status cannot be assessed based solely on one or a small number of molecular markers and these molecular markers together with EMT driving transcription factors should be assessed in conjunction with changes in cellular characteristics to define EMT. The guideline concluded that a combinatorial approach should be adopted to identify reliable EMT markers and their regulatory mechanisms.

To understand the multi-scale regulations of EMT, one could refer to how regulations of cell fate determination have been studied. The EMT process is crucial during the gastrulation stage of early embryo development for neural crest cells to delaminate and embark on differentiation towards their derivatives. During neural crest migration, after progressing through a sequence of common transcriptional traits, a series of binary decisions are made to determine subsequent cell fates^10^. The intertwined execution of EMT and cell fate commitment during neural crest development implies that the determination of epithelial and mesenchymal traits follows the concept of cell fate determination as depicted by the Waddington landscape proposed by Conrad Waddington in 1957. The entire landscape is featured with ridges and valleys indicating the barriers between each lineage and state with different intermediates to feature a plasticity spectrum. Therefore, reprogramming (direct or indirect) would require the system to overcome these barriers via the manipulation of various regulatory gatekeepers in multi-directionality^11^. Plasticity and fluidity of cell fate determination or EMT execution is thus dictated by the balance between different regulatory circuits governing the genome architecture, epigenetic modifications, transcription, translation, and post-translational modifications. Several reviews have nicely summarized the transcriptional^12^, translational^13^ control and post-translational modifications^14^ in EMT and will not be repeated here. In this Mini-Review, we will discuss studies looking at the regulation of EMT from the lenses of 3D genome organization and epigenetic reprogramming to explain how the plasticity along the EMT Spectrum could be established.

## 3D Genome 101

### The genome architecture in three dimensions

Following the emergence of chromatin conformation capture-based methods^15–19^, the studies of the chromatin architecture has broadened our understandings on how the genome follows hierarchical organization inside the nucleus. Harnessing the ease of mapping the 3D genome with these technology breakthroughs, the reorganization of the 3D chromatin conformation has been shown to influence cell identity during lineage-differentiation, cell cycle progression and could be dysregulated in diseases^20–24^.

**Fig. 2:**
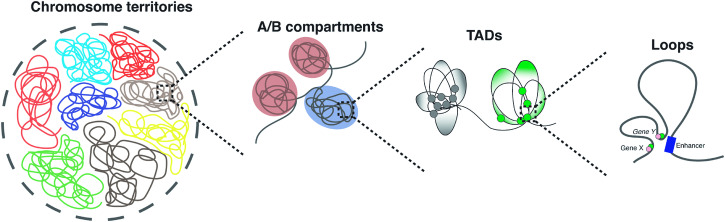
Hierarchical order of the 3D genome. The 3D genome in the nucleus is organized into hierarchical units of chromatin, with the formation of chromosomal territories (CTs) at the chromosomal resolution, where each chromosome occupies a distinct and non-overlapping area. Within CTs, there are chromatin compartments, comprising A compartments with open euchromatin enriched in highly expressed genes, and B compartments with closed heterochromatin associated with repressed transcription regions. Further organization within the compartments involves topologically associated domains (TADs), stable units guiding genome folding and long-range regulation. The finest level, often involving kilobases resolution, consists of chromatin loops which establish interactions between distant promoters and enhancers, playing a pivotal role in directly regulating gene expression.

The 3D genome is organized in the hierarchical orders from chromosome territories, active/inactive (A/B) chromosomal compartments, topological associating domains (TADs), and chromatin interactions involving long- and short-ranged DNA looping. Our genome thus follows certain origami-like patterns in coordination with a variety of components, such as transcription factors, architectural proteins, chromatin regulators, and non-coding RNAs to modulate transcription. At the largest resolution, chromosomes preferentially form topological territories with limited intermingling between chromosomes^25^ (Fig. 2). The chromosome territories can be further sub-divided into 2 main compartments corresponding to different chromatin types^19^, namely A (active) and B (inactive). Within the compartments, specific loci are arranged in spatial proximity to each other thereby forming TADs^22^, architectural “stripes”^26^ and loops^27^.

### Chromatin conformation capture-based techniques

The 3D genome can be studied in a genome-wide fashion using proximity ligation-based chromatin conformation capture methods such as Hi-C 1.0, HiC 2.0, HiC 3.0, Micro-C and Pore-C^27–32^, which often requires deep sequencing to fully map out the chromatin structural features. Alternatively, protein- or gene-centric chromatin conformation capture could be applied to interrogate the 3D genome in a more efficient and cost-effective manner^18,33–39^. With the plethora of techniques made available to map the 3D genome architecture, it is essential that one understands the information output of each technique before applying it to an EMT model system.

In light of approaches that focuses on local chromatin conformation of a handful of genes, low throughput chromatin conformation capture methods could prove to be useful such as 3C, where the interaction frequency between two or more interacting regions could be quantified^24,40^. In addition to query specific one-one interactions between two genomic loci, circularised chromatin conformation capture (4C) to look at the chromatin interactions originating from one loci^41,42^, or across many target loci along a continuous genomic region in chromosome conformation capture carbon copy (5C)^16^.

Besides ligation-based techniques, there are other alternatives to capture the 3D genome architecture, which includes genome architecture mapping (GAM)^43^, split-pool recognition of interactions by tag extension (SPRITE)^44^ and chromatin interaction analysis via droplet-based and barcode-linked sequencing (ChIA-Drop)^45^. In addition, there were two recent techniques that allowed the capture of chromatin conformation without the need for formaldehyde prefixing, namely DNA adenine methyltransferase (Dam)C^46^ and chemical-crosslinking assisted proximity capture (CAP-C)^47^. In essence, there are several methods available to map the 3D genome, as nicely summarized by previous reviews^48–50^, ranging from a high-throughput fashion such as HiC, SPRITE or GAM; to limited throughput techniques such as 3C, 4C and 5C which are capable of assessing specific enhancer-promoter interactions.

## Reconstructing the 3D genome in EMT models

The regulation of EMT from the epigenomic perspective has been elucidated from several recent studies. It is now clear that the transitional phases of EMT occurs together with complex changes at the epigenomic landscape^7,51–54^. During development, the expression of EMT genes have been shown to be regulated via both histone modifications and the local enhancer-promoter looping^55^. Similar regulatory mechanisms to the EMT genes have also been shown to exist in cancer cells^51,52,56,57^. The 3D genome hierarchical orders, together with histone modifications and local DNA looping, establishes the architectural backbone of the genome in response to any functional ques during EMT or cell fate determination during development and in cancer cells^53,58^.

### Higher order organizations in chromatin domains

**Fig. 3:**
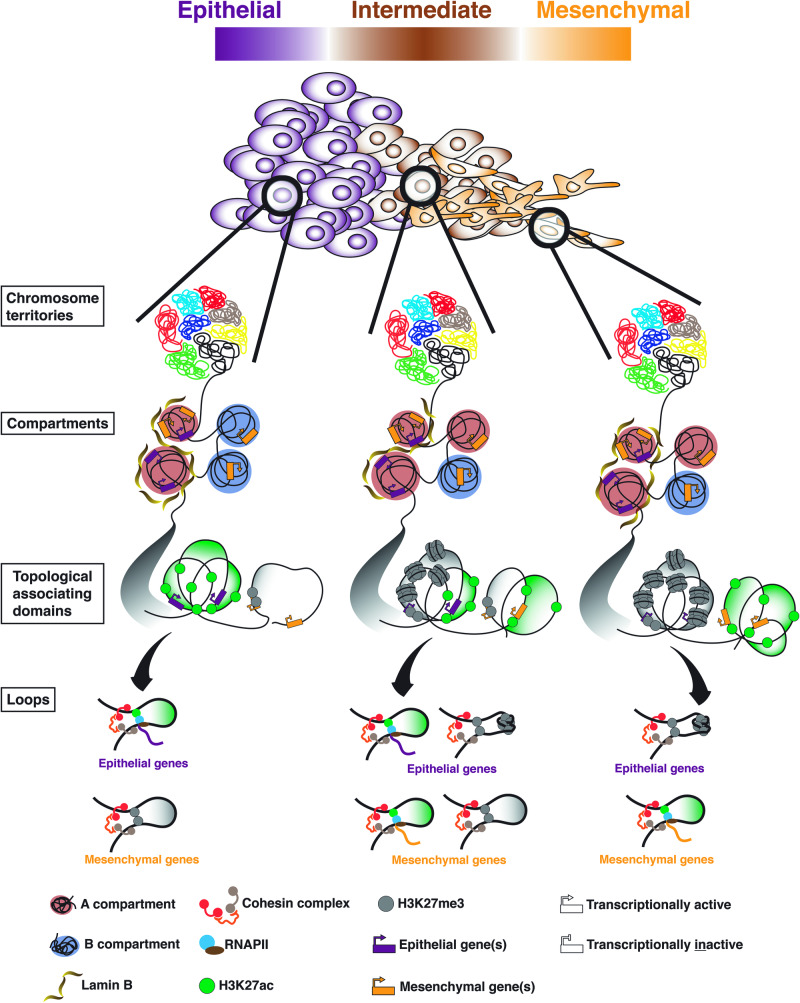
Illustration of the dynamic changes in the hierarchical organization of the 3D genome across different EMT transitional phases along the spectrum. The transition from an epithelial to mesenchymal state is depicted from left to right in the illustration. The hierarchical orders of the 3D genome organization, viewed from larger to smaller resolution (top to bottom) — chromosome territories, chromatin compartments, TADs and DNA loops —are indicated on the left. At the order of chromatin compartments, the eLADs are shown to be involved in the formation of new inactive chromatin compartments as the cells becomes mesenchymal. While the TAD borders remain relatively conserved between the EMT, the dynamic changes in histone modifications and chromatin conformation within the TADs are crucial in transcriptional regulation of EMT genes. Lastly, the formation of EMT-TF centric enhancer-promoter interactions, via loop extrusion model, within the TADs is required for activation of EMT gene transcription.

Genome-wide analysis of the 3D genome organization in EMT has been very limited among the vast expanding literature body on EMT. Two studies^59,60^ have shed light on potential changes in the 3D genome organization during EMT, which could occur at the nuclear periphery. These studies underscores the significance of large organized chromatin K9 modifications^61^ (LOCKs) and nuclear lamina-associated domains^62,63^ (LADs) in playing a contributory role in the functional reorganization of the 3D genome within the nucleus. Notably, both LOCKs and LADs are typically transcriptionally repressed chromatin regions while often in proximity to the nuclear lamina^61,63^. The studies explore their involvement and events leading to the transcriptional activation of EMT-related genes during the EMT process. Using chromatin immunoprecipitation followed by genomic DNA microarray analysis (ChIP-chip) in TGF-β-induced AML12 mouse hepatocytes, the study by McDonald et al.^59^ has implicated extensive chromatin reprograming during EMT at the LOCKs, which are large (100 kb–5 Mb), non-repetitive heterochromatin domains enriched in H3K9me2^59^. In this model, there is a global reduction in the heterochromatin mark H3 Lys9 dimethylation (H3K9me2), accompanied by the increase in euchromatin marks H3 Lys4 trimethylation (H3K4me3) and mark H3 Lys36 trimethylation (H3K36me3). The quantitative reduction in H3K9me2 is due to LSD1 demethylation the LOCKs, leading to the recruitment of H3K4me3 within the LOCKs and the enrichment of H3K36me3 at the boundaries of the LOCKs. This enrichment of H3K36me3 at the boundaries leads to the transcriptional activation of genes with EMT-related functions. Further studies would need to be conducted to assess whether these LOCK-specific H3K4me3 changes could trigger the release from the nuclear lamina, prior to transcriptional activation. While lamin A/C’s functional role in gene regulation is well-established, it predominantly associates with euchromatic regions, whereas lamin B is primarily linked to heterochromatin^64^. Interestingly, the presence of lamin B1 within euchromatic regions follows a dynamic nature. Utilizing a TGF-β induced EMT model in non-transformed mouse mammary gland epithelial cell line (NMuMG)^60^, the authors discovered the existence of euchromatin LADs (eLADs), which is formed as lamin B1 engages with euchromatin regions. These eLADs exhibit transcriptional activity in the active compartments throughout the EMT process (Fig. 3). Intriguingly, as the cell becomes mesenchymal, there is a surge in transcriptionally inactive eLADs within the inactive compartments. This increase corresponds to newly formed eLADs, disputing the notion of eLADs merely shifting between active and inactive compartments. Moreover, the study identified a correlation between the enrichment of lamin B1 and the strength of TAD boundaries in active chromatin compartments, highlighting the possible role of lamin B1 as an architectural protein in mediating the establishment of new genome architecture during EMT.

These two studies support the notion that LADs and possibly the interface between the heterochromatin and euchromatin at the compartment level serve as the crucial chromatin structural organizing sites.

### Dynamics of active and inactive compartments in EMT

**Fig. 4:**
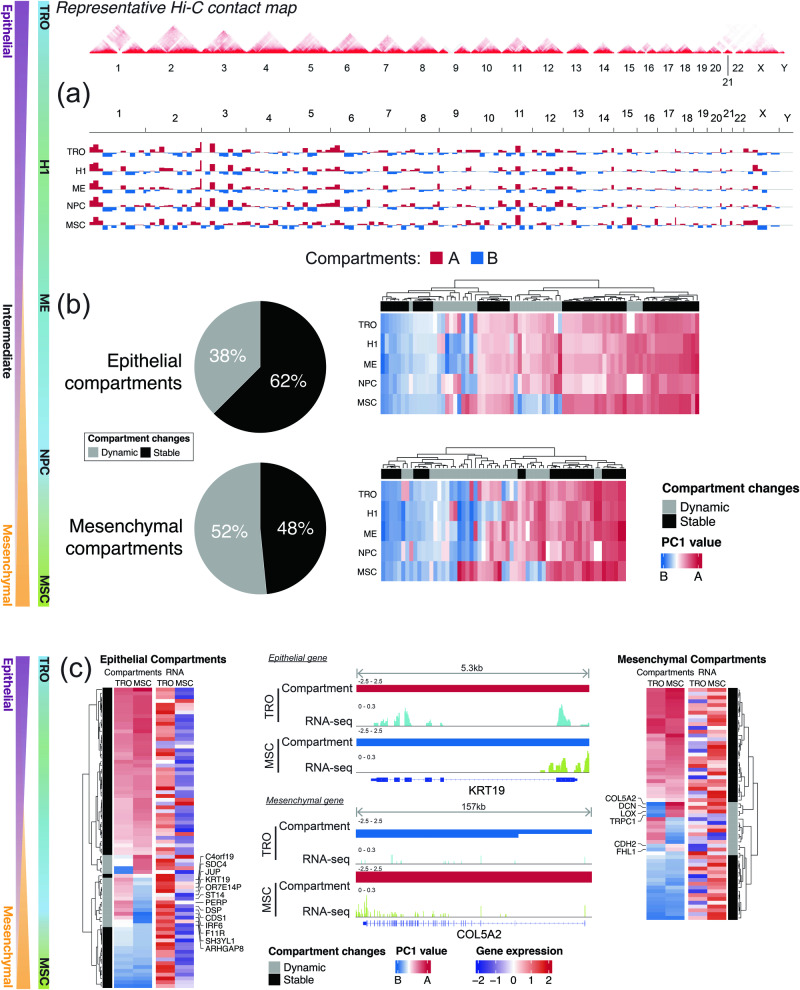
Illustration of 3D genome compartment dynamics of EMT in stem cell differentiation and cancer models. Stem cell model was derived from the differentiation of H1-hESC cells. H1 – H1 hESC, ME – mesendoderm, TRO – trophoblast-like cell, NPC – neural progenitor cell and MSC – mesenchymal stem cell. Cell lines are arranged by their EMT score. **a** Genome-wide view of A and B compartments, colored red and blue respectively, in H1-hESC and its differentiated cell lines. **b** Pie chart depicting the distribution of dynamic (gray) and stable (black) compartments across the cell lines at both epithelial and mesenchymal compartments. Heatmap representation of the compartment PC1 values are shown on the right of each pie chart. **c** Heatmap representation of PC1 and expression values of epithelial (right) and mesenchymal (left) genes in TRO (EMT score = −0.17) and MSC (EMT score = 0.62). IGV Snapshots of an epithelial (KRT19; 5.3 kilobase locus) and mesenchymal (COL5A2; 157 kilobase locus) gene demonstrating concordant compartment and expression dynamics is shown in the middle.

The comprehension of compartment changes during EMT remains incomplete due to limited available studies. However, insights into the dynamics of 3D genome compartments during EMT can be inferred from observations in two different cell line models, namely (i) stem cell differentiation^65^ and (ii) cancer EMT^53^. Both models employed the Hi-C technique to interrogate the 3D genome organization. One model focused on human embryonic stem cells and their derivative differentiated cell lines^65^, while the other involved cancer cells representing various transitional phases of EMT^53^. In both the stem cell differentiation model (Fig. 4a) and the cancer EMT cell line model, the A/B compartments housing EMT genes displayed dynamic changes and conservation across different EMT transitional phases. This underscores the role of A/B compartments in regulating stem cell differentiation, as previously reported^65,66^, which is also evident in the epithelial and mesenchymal compartments. However, it’s important to note that the magnitude of change in the compartments doesn’t necessarily correspond to a similar change in the expression of specific epithelial/mesenchymal genes (Fig. 4b). Only a subset of EMT genes exhibit such concordant dynamics between compartments and gene expression (Fig. 4c). For instance, classical epithelial and mesenchymal genes, including *KRT19*, *LOX*, *COL5A2*, and *CDH2*, exhibited concordant compartmental switch between EM states in both the stem cell differentiation (Fig. 4c) and cancer EMT cell line models^53^. Taken together, the two models collectively indicate distinct regulation patterns for epithelial and mesenchymal genes at the 3D genome level. This distinction was further elucidated in the cancer EMT model, where epithelial genes were progressively repressed along the EMT spectrum, characterized by increased H3K27me3 binding and cell-type specific changes in chromatin interactions. In contrast, the regulation of the mesenchymal genes was more closely associated with the changes in local chromatin conformation, where contact frequency within the mesenchymal TADs were shown to increase along the EMT spectrum^53^. The reprogramming of histone modifications within the TADs was also evident in an TGF-β induced EMT model in lung cancer cells, dynamic changes of specific histone H3 lysine 27 acetylation (H3K27ac)-marked enhancers mainly occur within pre-existing TADs in epithelial cells to activate mesenchymal genes^67^. This underscores the inherent and distinct regulation of epithelial and mesenchymal genes in EMT at the 3D genome level.

### Promoter-enhancer interaction

The activation of gene transcription relies on the crucial physical contact between enhancers and promoters^68^, often necessitating DNA looping, which can span distances ranging from 1 kb to several megabases^69–73^. Studies on chromatin conformation changes during EMT have employed the 3C technique to investigate the regulation of local promoter and enhancer interactions for specific genes^55,56,74^. For instance, in the *Drosophila* embryo, Snail represses the transcription of 2 dorsal-ventral patterning genes *sog* and *brk* by blocking the formation of promoter-enhancer interactions when Snail is bound to the distal enhancer^75^. In the mouse epithelium, the epithelial-specific transcription factor (TF) GRHL2 regulates the looping between the promoter and the enhancer of *Cdh1* at intron 2 to maintain epithelial integrity^55^. During TGF-β induced-EMT in NMuMG cells, the expression of E-cadherin was mediated by DNA looping between the transcription start site (TSS) of *Cdh1* to the enhancers bound by GRHL2 and HNFα^74^. These findings confirm that short-range local promoter-enhancer interactions, regulated by the TF complex via DNA looping, play a crucial role at EMT gene loci.

### Cohesin-mediated loop formation

The stability of promoter-enhancer looping relies on the cohesin complex^76,77^. The cohesin complex consists of SMC1, SMC3, RAD21 and SA1 or SA2 proteins^78^. The loading and unloading of cohesin from chromatin are further controlled by the complexes, NIPBL-MAU2 and WAPL-PDS5^79–81^. The formation of loops requires cohesin intrinsic ATPase activity to load and slide along DNA as proposed in a loop extrusion model. The idea of loop extrusion was first described in studies of axolotl lampbrush chromosomes in 1882 by Walther Flemming^82^. Subsequently, the loop extrusion hypothesis was further explored^83–85^ and nicely summarized in a recent review^86^. Recent studies tested the loop extrusion model and this was suggested by ChIP-seq data in ATP-depleted cells where RAD21 binding was stronger at NIPBL loading sites while weaker at CTCF anchor sites^26^. Although ATP-independent loops are also formed in the absence of cohesin translocation along DNA, it was observed that cohesin-mediated loop formation tends to favor long-range functional interactions between enhancer and their cognate promoters^26^. Additionally, the loop extrusion model was also shown by cohesin depletion by Schwarzer et al. in a mouse model^87^ and through real-time imaging the formation DNA loops by *Saccharomyces cerevisiae* condensin complex further validated the model^88^.

In an EMT model of breast cancer cells, reduced binding of the cohesin complex component RAD21 at the loci of *TGFB1* and *ITGA5* leads to a loss of intrachromosomal interactions that allows recruitment of transcriptional machinery to the promoters of the mesenchymal genes^56^. In a SNAI1-inducible EMT model of ovarian cancer cells, RAD21 was found to be selectively depleted from the distal enhancer sites of the epithelial genes *ERBB3* and *PERP*. This selective depletion of RAD21 binding was correlated with the various states along the EMT Spectrum^51^. A recent study further suggested that another cohesin complex component SMC1A could bind to the *SNAI1* promoter to regulate its transcription^89^ pointing to a possible feedback regulation between cohesin and the EMT TF machinery. However, it is unclear whether these cohesin complex binding events would have impact on the local DNA looping since there were no chromatin conformation data available. Nevertheless, these studies provided us with a zoomed in glance at the local DNA looping interactions of selected EMT gene loci.

## The epigenomic accessibility landscape in EMT models

The epigenomic landscape in EMT models, intertwined with 3D genome architecture, significantly influences chromatin accessibility. The dynamic phase transition along the EMT spectrum mimics the energy gradient which could be segregated according to the epigenetic changes^90^ to drive cellular plasticity. Many of the EMT TFs are known to recruit epigenetic modifiers affecting downstream targets^91^, with pioneer-factor like TFs^52^ demonstrating modulation of EMT plasticity through nucleosome remodeling. These EMT TFs themselves are often epigenetically regulated thus forming a reciprocal feedback control. Through a combined approach of pharmacological and CRISPR interference screens coupled with synthetic genetic tracing of EMT in lung cancer cells, several chromatin remodelers, writers, and readers were found to govern over phenotypic plasticity via functional antagonism^92^. Through exploiting the EMT plasticity facilitated by Polycomb repressive complex 2 (PRC2) inhibition in lung cancer cells, Serresi et al. uncovered functional antagonism between several chromatin modulators occurring at active chromatin regions during EMT^92^. Pivotal to plasticity, the state of bivalent chromatin regulated by PRC2 is particularly crucial in governing the epigenomic landscape of EMT^93^. Bivalency in chromatin characterization was first coined by Bernstein and colleagues to describe the conflicting combination of active and inactive histone marks observed at same genomic regions^94^. Earlier studies have shown that PRC2 subunits co-orchestrate with EMT TFs during the epigenetic reprogramming of EMT^95,96^. In a breast cancer cell model, PRC2 methyltransferase enhancer of zeste homolog 2 (EZH2) cooperates with SOX4 in TGF-β-induced EMT regulating lysine 27 on histone H3 (H3K27me3) modifications at the promoters of EMT genes^41^. In another *TWIST1*-induced EMT model in immortalized human mammary epithelial cells (HMLE), EZH2 is required for EMT execution, and the maintenance of bivalent chromatin marked by H3K27me3^96^. In this study, switching in between active histone mark (H3K4me3) to the bivalent/repressive mark (H3K27me3) is found at the promoter of EMT genes. Following *TWIST1*-induced EMT, there are 75 genes presenting promoter switching from H3K4me3 to H3K27me3 and 144 genes presenting promoter switching from H3K27me3 to H3K4me3. In another model of ovarian cancer cells, the loss of the epithelial-specific TF *GRHL2* causes the switch of the chromatin states at the promoters of known EMT signature genes^97^ during EMT. Approximately 5% of the active chromatin switched into poised/bivalent or PRC2-repressed chromatin at the GRHL2 binding sites^52^. These findings highlight the important role of the bivalent chromatin associated with the PRC2-related H3K27me3 modification during EMT.

What is intriguing is the fact that there are very few overlapping genes with a concordant H3K27me3 modification change following EMT induction between these studies. For example, the pioneer TF of B-cell specification and commitment gene *Ebf1* is the only gene showing concordance in these two studies with the loss of H3K27me3 mark at its promoter following EMT. Another overlapping gene *Lgr6* is upregulated with the loss of H3K27me3 mark in the Tiwari study but is found to harbour the promoter switch from H3K4me3 to H3K27me3 in the Malouf study. Among a selected panel of EMT signature genes showing the switch from the active state to the poised/bivalent or repressed states at the promoter in the Chung study, three genes (*ESRP1*, *GRHL2*, *LAD1*) are concordant with those switching from H3K4me3 to H3K27me3 at the promoter in the Malouf study. A recent study might be able to provide some clues for this discrepancy that these H3K27me3 enriched PRC2 sites are further classified according to different regulatory mechanisms^98^. Since PRC2 preferentially binds to the CpG islands (CGI) at the promoters, this study explores the genome-wide PRC2-occupied CGI (PRC2^+^-CGI) across 16 TCGA cancer types. Approximately 50% of H3K27me3 enriched PRC2^+^-CGI promoters in at least one cancer type are hypermethylated with down-regulation of transcription. There are about 35% of PRC2^+^-CGI promoters which are transcriptionally up-regulated with high chromatin accessibility in at least one cancer type. These upregulated PRC2^+^-CGI genes (not the hypermethylated GCIs) are enriched in the EMT pathway in many cancer types including basal breast cancer (Basal BRCA) and lung cancers. A small subset of mesenchymal genes (*CDH2*, *DKK1*, *SERPINE2*, *MATN3, APLP1, CXCL1*, *FOXC2*, *BMP1*) in Basal BRCA has been validated in this PRC2^+^-CGI pool with *SERPINE2* and *CXCL1* being the 2 most commonly overlapping genes found in 7 and 6 cancer types, respectively. This cancer-specific pattern of regulation at the PRC2 target sites is mediated by the cooperation with specific TFs binding to the distal enhancers to confer tissue specificity. This creates the promoter-enhancer interactions to allow cancer-specific TFs from distal enhancers to affect the chromatin accessibility at proximal promoters to regulate different sets of EMT genes. Findings from these studies reveal a versatile landscape of epigenomic regulation of EMT and the complexity of context-dependent regulation indicates that there might not exist a universal master regulator. This further provides a mechanistic ground as why the definition of EMT cannot rely on one or a small number of molecular markers^5^.

## Heterogeneity and dynamic genome organization of EMT

A recent study using live-cell imaging to trace single cells in a TGF-β induced EMT model in the lung cancer A549 cells further confirms that the transition dynamics proceeds through two parallel paths^99^. The challenges ahead in the EMT field would lie in the delineation of the different trajectories undertaken by each single cell that are at different phases of the EMT spectrum. Single cell transcriptomic profiling has shown that EMT is not a linear but highly variable process that branches into various trajectories upon induction^9,100^. The complexity is further amplified by the regulatory controls present at multidimensional levels. While single cell transcriptomic profiling has enabled us to look at EMT from an individualistic perspective, the chromatin regulation of the transcriptional activity of EMT genes in concert with TFs is still largely uncharted at a single cell resolution. By using Assay for Transposase-Accessible Chromatin using sequencing (ATAC-seq) in single cells, germline stem cells (GSCs) have been clustered into epithelial-like and mesenchymal-like states that constitute into an EMT spectrum^101^.

With advances in single cell multi-omics sequencing, simultaneous profiling of the transcriptome and epigenome paves the way to bridge the link between epigenetic controls leading to transcriptional events that constitute the heterogeneity of EMT. For instance, several techniques have been developed to capture both mRNA with chromatin accessibility from the same cell^102–104^. This could provide information to correlate between chromatin accessibility and gene expression in EMT models, as well as elucidate the possible TFs that orchestrate the accessibility changes at the EMT genes at different EMT states. Taken into account that EMT-TFs could also regulate their downstream genes through DNA methylation, a technique developed by Gu et al. that captures information on both DNA methylation and accessibility from a single cell would have potential to provide further insights on the regulatory mechanisms of EMT-TFs along the different transitional phases during EMT^105^.

To decipher the role of 3D genome in the regulation of EMT at the single cell resolution, technical breakthroughs are crucial to accelerate discovery. The techniques available at the moment for generating single cell 3D genomes are either of low throughput^53^ or lack the capability to simultaneously characterize the EMT states of each single cell via the expression of epithelial/mesenchymal markers at the high throughput setting^23,106–109^.

In conjunction to scHi-C, computational methods which maximises for the sparseness observed in most scHi-C data are also crucial. Current computational methods to enhance scHi-C datasets include unsupervised embedding^110,111^, random-walk-based imputation^112^ or hypergraph representation learning of contact maps^113^. Despite the sparsity of scHi-C contacts, it was shown that at the single-cell level, the chromosomal conformation at *VIM* was able to delineate single-cells by their EMT states, as determined by fluorescence measure of the EMT protein markers^53^. With the advent of more technological breakthroughs, sophisticated seqFISH^114,115^ techniques such as DNA and intron seqFISH+ could enable us to elucidate the dynamics of nuclear organization and transcriptomics during EMT through spatial genomics in a temporal fashion, aspects that are largely obscured in current single-cell techniques (Box 1).

Box 1 Outstanding questions and challenges

- How do we map the diverse trajectory paths of master EMT-TFs occurring on a multi-omics scale – from epigenome, transcriptome, to proteome?
  - EMT is a multistep process orchestrated by a plethora of EMT-TFs^4,5,127^.
  - Evidence of transitional EMT states have been described in in vivo models^7,128^ with different modes of regulation undertaken by EMT-TFs.
  - Perturbational single cell omics could serve as a useful tool in interrogation of diverse EMT regulation networks^129^.
  - However, context is critical as cell-type specific EMT regulation needs to be considered.
  - Hitherto, single-cell sequencing studies of EMT in cancer (Table S1) are mainly focused on the transcriptome or epigenome.
  - The lack of simultaneous capture of epigenome, protein, and mRNA expression at single-cell depth in these studies may not adequately define the EMT states.
- How does the 3D genome contribute to EMT in a spatiotemporal manner, and to what extent?
  - In the context of dynamic and preformed loops, what are the implications that might arise from such chromatin conformation models during the transition from epithelial to mesenchymal state?

## Future perspectives

Currently, we discussed the EMT spectrum in the context of transcriptional signature, there are other functional subtypes of the spectrum, including the ameboid state^116^. Although EMT occurs in a context-dependent manner, therein lies core regulatory networks present in both physiological and pathological conditions which are previously summarized in recent reviews^4,117^, and the checkpoints at each transitional phase of the EMT spectrum could be governed by different EMT-TFs of the core regulatory networks (EMT-inducers or EMT-gatekeepers)^7,52,118,119^. Therefore, the use of chromatin conformation techniques in future EMT models, such mouse models^7,60^ or cell lines^53,67,120^, should be considered carefully and as recommended by the latest TEMTIA guidelines^5^. In a comprehensive approach to map the 3D genome changes between transitional phases along the EMT spectrum, HiChIP or ChIA-PET could be used to elucidate the EMT-TFs-centric chromatin interactions. By mapping the diverse EMT-TFs trajectory paths in a spatiotemporal manner, it would be possible to identify epigenomic events which predate the downstream activation of EMT transcriptional programs at various transitional phases of EMT. This could facilitate our comprehensive understanding of how the 3D genome and the EMT-TFs co-orchestrate the plasticity landscape. Given the transitional and continuous nature of the EMT Spectrum^7,97,121–126^, it is crucial to delineate the regulatory landscape of subpopulations of different EMT states under different context. The heterogeneity and dynamics of EMT regulatory epigenome needs to be answered via single cell-omics platforms with close collaborations with multidisciplinary computational and mathematical modelling expertise. These efforts will help decipher how the plasticity occurs during EMT under different context to guide the future development of translational applications of the EMT concept in diseases.

### Supplementary information

Supplementary information
