# Supplementary material for: Regulating epithelial-mesenchymal plasticity from 3D genome organization
Source: Commun Biol. 2024 Jun 20;7:750. doi: 10.1038/s42003-024-06441-w (PMC11190238; doi:10.1038/s42003-024-06441-w)
Supplement: Supplementary file 1 — Supplementary information [file 42003_2024_6441_MOESM1_ESM.pdf]

Supplementary information

Table S1

**Cancer/Tissue**	**Brief description**	**Source**	**Single-cell sequencing**
colorectal, gastric, nasopharyngeal, SCC, ovarian, PDAC and breast	Integrative analysis of 266 tumors across 8 different cancer types	Cook and Vanderhyden 2022[1]	scRNA-seq
Bladder cancer	scRNA-seq and scATAC-seq on 13 tumors	Wang, Mei et al. 2022[2]	scRNA-seq scATAC-seq
MCF10A, breast	TGF-β1 in MCF10A cells	Deshmukh, Vasaikar et al. 2021[3]	scRNA-seq
LUAD	scATAC-seq profile in lung adenocarcinoma mouse model	LaFave, Kartha et al. 2020[4]	scRNA-seq scATAC-seq
A549, lung; DU145, prostate; MCF7, breast; and OVCA420, ovarian	Data from Cook et al., 2020	Sha, Wang et al. 2020[5]	scRNA-seq
A549, lung; DU145, prostate; MCF7, breast; and OVCA420, ovarian	TGFB1, EGF, and TNF in various cell lines	Cook and Vanderhyden 2020[6]	scRNA-seq
HNSCC	Primary & metastatic tumors	Puram, Tirosh et al. 2017[7]	scRNA-seq

References

1 Cook, D. P. & Vanderhyden, B. C. Transcriptional census of epithelial-mesenchymal plasticity in cancer. *Science Advances* **8**, eabi7640 (2022). https://doi.org:doi:10.1126/sciadv.abi7640

2 Wang, H. *et al.* Single-Cell Analyses Reveal Mechanisms of Cancer Stem Cell Maintenance and Epithelial–Mesenchymal Transition in Recurrent Bladder Cancer. *Clinical Cancer Research* **27**, 6265-6278 (2022). https://doi.org:10.1158/1078-0432.Ccr-20-4796

3 Deshmukh, A. P. *et al.* Identification of EMT signaling cross-talk and gene regulatory networks by single-cell RNA sequencing. *Proc Natl Acad Sci U S A* **118** (2021). https://doi.org:10.1073/pnas.2102050118

4 LaFave, L. M. *et al.* Epigenomic State Transitions Characterize Tumor Progression in Mouse Lung Adenocarcinoma. *Cancer Cell* **38**, 212-228.e213 (2020). https://doi.org:10.1016/j.ccell.2020.06.006

5 Sha, Y., Wang, S., Bocci, F., Zhou, P. & Nie, Q. Inference of Intercellular Communications and Multilayer Gene-Regulations of Epithelial-Mesenchymal Transition From Single-Cell Transcriptomic Data. *Front Genet* **11**, 604585 (2020). https://doi.org:10.3389/fgene.2020.604585

6 Cook, D. P. & Vanderhyden, B. C. Context specificity of the EMT transcriptional response. *Nat Commun* **11**, 2142 (2020). https://doi.org:10.1038/s41467-020-16066-2

7 Puram, S. V. *et al.* Single-Cell Transcriptomic Analysis of Primary and Metastatic Tumor Ecosystems in Head and Neck Cancer. *Cell* **171**, 1611-1624.e1624 (2017). https://doi.org:10.1016/j.cell.2017.10.044
